# *Tbp* and *Hprt1* Are Appropriate Reference Genes for Splenic Neutrophils Isolated from Healthy or Tumor-Bearing Mice

**DOI:** 10.3390/biomedicines12112571

**Published:** 2024-11-10

**Authors:** Khetam Sounbuli, Ludmila A. Alekseeva, Aleksandra V. Sen’kova, Innokenty A. Savin, Marina A. Zenkova, Nadezhda L. Mironova

**Affiliations:** 1 Institute of Chemical Biology and Fundamental Medicine SB RAS, Lavrentiev Ave., 8, Novosibirsk 630090, Russia; khetam.sounbuli.edu@gmail.com (K.S.); alekseeva.mila.23@yandex.com (L.A.A.); senkova_av@niboch.nsc.ru (A.V.S.); savin_ia@niboch.nsc.ru (I.A.S.); marzen@niboch.nsc.ru (M.A.Z.); 2 Faculty of Natural Sciences, Novosibirsk State University, Pirogova St., 1, Novosibirsk 630090, Russia

**Keywords:** neutrophils, splenic neutrophils, spleen-derived neutrophils, activated neutrophils, reference gene, house-keeping gene, gene expression stability, RT-qPCR

## Abstract

**Background**/**Objectives**: Neutrophils have recently gained significant attention due to their heterogeneity in tumor settings. The gene expression profiles of neutrophils from different tumor types are of great interest. Murine splenic neutrophils reflect the immune status of the organism and could be a source of tumor-associated neutrophils in tumor-bearing mice. However, information about appropriate reference genes for RT-qPCR analysis of murine neutrophils in the literature is lacking. The aim of this study was to identify stably expressed reference genes in murine splenic neutrophils. **Methods**: Bone marrow- and spleen-derived neutrophils were isolated from healthy C57Bl/6 and CBA/LacSto mice. Spleen-derived neutrophils were isolated from mice with Lewis lung carcinoma (LLC) and drug-resistant lymphosarcoma (RLS_40_). RNA was isolated and used for RT-qPCR analysis of 10 selected reference genes. Analysis of reference gene stability was performed using four different algorithms (BestKeeper, NormFinder, geNorm, ΔCt method), and comprehensive ranking was constructed using RefFinder. **Results**: The Ct values for the reference genes were in the range of 16.73–30.83 with the highest expression levels observed for B2m and the lowest for Sdha. Differences in the stability ranking performed by different algorithms were observed; however, the overall ranking of the studied reference genes was as follows, from most to least stably expressed: *Tbp*, *Hprt1*, *Ywhaz*, *B2m*, *Gapdh*, *Actb*, *Sdha*, *Eef2*, *Rack1*, and *Rpl13a*. Using *Tbp* or *Rpl13a* for RT-qPCR data normalization significantly affected the interpretation of target gene expression. **Conclusions**: *Tbp* and *Hprt1* are recommended reference genes for murine splenic neutrophils regardless of their activation status.

## 1. Introduction

Neutrophils are the key cells of the innate immune system and the first line of defense against pathogens. They represent the most abundant leukocyte type in human blood and the second most abundant in murine blood [1]. Recently, neutrophils were shown to play an important role in cancer settings [2]. They were shown to gain pro- or anti-tumor phenotypes that contribute to tumor development or elimination, respectively [3,4,5]. Neutrophil polarization in the tumor microenvironment (TME) is a complicated process influenced by several tumor-derived factors [4]. Tumor-associated neutrophils exhibit anti-tumor activity through direct cytotoxicity via the production of reactive oxygen and nitrogen species (ROS and RNS) and antibody-dependent cellular cytotoxicity, and the most effective mechanism is the activation of immune cell cytotoxicity in the TME [6]. The pro-tumor phenotype is exhibited by creating an immunosuppressive TME and supporting tumor angiogenesis [4]. Moreover, neutrophil extracellular traps (NETs) support tumor cell metastasis [7]. Recent studies have focused on the role of neutrophils in cancer therapy, identifying neutrophils as the determinant component of the success of immunotherapy against cancer [8,9].

In the last few years, splenic neutrophils have gained considerable attention in cancer studies. Splenic neutrophils could represent the actual phenotype of neutrophils in the organism because the spleen is considered one of the primary sites of migration for neutrophils [10]. Although most splenic neutrophils result from clearance, recent reports have shed light on splenic granulopoiesis and spleen-residential neutrophils [11,12,13]. Splenic neutrophils were reported to save their functionality and response to activation [14,15]. Many studies reported their role in physiological and pathological conditions [12,16,17,18,19,20].

In the case of tumors, splenic neutrophils can display tumor-associated neutrophil phenotypes, which makes their gene expression profiles of great interest [21,22,23,24]. The splenic neutrophil gene expression profile in a murine model of leukemia was significantly altered to a pro-tumor profile [22]. In breast cancer models, splenic neutrophils develop a glycolytic profile and create a glucose-deprived microenvironment that inhibits antitumor T-cell activity against tumor cells [24]. Whether these findings regarding the splenic neutrophil profiles in cancer can be extended to other cancer models remains to be proven; however, there is no doubt that splenic neutrophils are of great interest in tumor settings. These findings confirm the differential phenotype of splenic neutrophils compared with naïve bone marrow-derived neutrophils, making splenic neutrophils a representative population of in vivo activated neutrophils.

Reverse transcription quantitative PCR (RT-qPCR) is one of the most widely used methods for studying gene expression. In relative quantification, which is commonly used in RT-qPCR, the expression of a control gene, referred to as the reference gene, is used to normalize the expression of target genes. Reference genes are those believed to have stable expression with no variation (or minimal variation) in the studied sample set, belong to cellular maintenance pathways, are essential, and are conserved [25,26]. Currently, it is commonsense that different treatments, experimental conditions, or physiological processes, such as differentiation or activation, can affect the expression of reference genes in studied cells, leading to incorrect interpretations of RT-qPCR results [25,27,28,29,30,31].

The issue of reference gene selection is particularly important in neutrophils because neutrophils can actively change their transcriptome in response to minor environmental cues [32]. Moreover, since splenic neutrophils in tumor settings are activated in contrast to bone-marrow-derived neutrophils, and under cell activation, not all reference genes remain stable [28], it is important to select a reference gene whose expression is stable in naïve or resting neutrophils and still stable in activated splenic neutrophils. For example, B2m, a commonly used reference gene in neutrophils, is an immunity-related gene that can be regulated in a changed manner in activated versus naïve neutrophils [33].

Mathematical methods were developed to evaluate the expression stability of reference genes in different samples. These methods are integrated in different programs and web-based tools [34,35,36]. The most commonly used instruments or methods are BestKeeper [37], NormFinder [38], geNorm [39], ΔCt method [40], and RefFinder [34]. With the help of these instruments, many studies have identified optimal reference genes for different cell types and tissues [28,41,42,43,44,45,46,47,48,49,50]. However, to the best of our knowledge, there are no reports strictly aimed at the selection and validation of appropriate reference genes for murine splenic neutrophils. Therefore, the objective of our study was to identify appropriate reference gene(s) that could be used for murine neutrophils regardless of their activation status.

## 2. Materials and Methods

### 2.1. Mice

C57Bl/6 and CBA/LacSto (hereinafter called C57Bl and CBA) male mice were obtained from the vivarium of ICBFM SB RAS (Novosibirsk, Russia). Mice were 3–4 months old. The body weight of mice was about 20–25 g. Mice were housed in plastic cages in standard daylight conditions. Water and food were supplied ad libitum. All animal procedures were conducted in strict compliance with the guidelines for the proper use and care of laboratory animals (ECC Directive 2010/63/EU). The experimental protocols were approved by the Committee on the Ethics of Animal Experiments with the Institute of Cytology and Genetics SB RAS (ethical approval number 49 from 23 May 2019).

### 2.2. Tumor Strains

The LLC tumor strain was a generous gift from Dr. N. Popova (Institute of Cytology and Genetics SB RAS, Novosibirsk, Russia). Drug-resistant murine lymphosarcoma (RLS_40_) was obtained from the cell collection at the Institute of Chemical Biology and Fundamental Medicine SB RAS.

### 2.3. Tumor Transplantation and Design of Animal Experiments

The LLC strain was routinely propagated in vivo by intramuscular transplantation. Freshly dissected tumors were homogenized in saline buffer and cells were applied onto a 70 μm cell strainer (Corning, Glendale, AZ, USA). The cells then were centrifuged on a layer of lymphocyte-separation medium (MP Biomedicals, Santa Ana, CA, USA) at 300× *g* for 10 min. Then, the cells at the upper interface of the separation medium were harvested and washed with PBS 2 times at 250× *g*. After that, the cells were resuspended in saline buffer and C57Bl mice (n = 20) were injected intramuscularly (i.m.) into the right thighs with LLC cells (10^6^ cells/mouse) in 0.1 mL of saline buffer. On day 15 after tumor transplantation, mice were euthanized under isoflurane anesthesia. A gas mixture containing 3% isoflurane and 97% air was used at a flow rate of 2 L/min. After, the spleens were harvested from mice with a tumor node size ≥ 1 cm^3^ to isolate splenic neutrophils as described below.

RLS_40_ solid tumors were induced in CBA mice (n = 40) by intramuscular (i.m.) injection into the right thighs of tumor cells (10^6^) in 0.1 mL of saline buffer. On day 21, the mice were divided into two groups depending on the size of tumor nodes: RLS_40_^High^ mice with a tumor node size ≥1 cm^3^ (n = 23) and RLS_40_^Low^ mice with a tumor node size ≤ 0.1 cm^3^ (n = 17). The mice were euthanized, and the spleens were harvested to isolate splenic neutrophils as described below.

### 2.4. Bone Marrow Cell (BMC) Isolation

BMC suspension of healthy mice was prepared as described in [14]. Briefly, an insulin U-100 syringe was used to flush the femur and tibia with RPMI 1640 (ThermoFisher Scientific, Waltham, MA, USA) supplemented with 10% FBS (BioFroxx, Einhausen, Germany), 1% antibiotic-antimycotic solution (MP Biomedicals, Santa Ana, CA, USA), and 2 mM EDTA (MP Biomedicals, Santa Ana, CA, USA), followed by red blood cell lysis and a two-step wash [14]. The BMC yield was (0.38 ± 0.06) × 10^8^ cells for CBA mice and (0.66 ± 0.19) × 10^8^ cells for C57Bl mice.

### 2.5. Splenocyte Suspension Preparation

Spleens were harvested from healthy and tumor-bearing mice, and splenocyte suspensions were prepared as described in [14]. The spleen suspension was prepared in 1 mL of RPMI 1640 supplemented with 10% FBS, 1% antibiotic–antimycotic solution, and 2 mM EDTA. The spleen was homogenized mechanically with the thumb rest side of a syringe’s plunger. After homogenization, the cell suspension was filtered using a 70 μm cell strainer (Corning, Glendale, AZ, USA), and a two-step wash with PBS was conducted after the lysis of red blood cells [14]. For healthy mice, the splenocyte population was about (0.78 ± 0.53) × 10^8^ and (0.93 ± 0.74) × 10^8^ cells per mouse for CBA and C57Bl, respectively. For tumor-bearing mice, the yield was (1.32 ± 0.50) × 10^8^ cells per mouse in the LLC group, (0.74 ± 0.22) × 10^8^ cells per mouse in the RLS_40_^Low^ group and (0.91 ± 0.11) × 10^8^ cells per mouse in the RLS_40_^High^ group.

### 2.6. Neutrophil Isolation

Bone marrow-derived neutrophils and splenic neutrophils were isolated from BMC or spleen suspensions from pooled samples of five bone marrows or spleens from each group using immunomagnetic positive selection as previously described [14]. Trypan blue exclusion assay was used to assess cell viability (Shanghai Macklin Biochemical Technology Co., Ltd., Shanghai, China). A Goryaev chamber and an Axiostar plus microscope (Zeiss, Munich, Germany) were used to measure the neutrophil yield and viability. For healthy CBA mice, the neutrophil yield was (9.31 ± 0.31) × 10^6^ and (2.44 ± 0.11) × 10^6^ cells for bone marrow and spleen, respectively, and for healthy C57Bl mice, it was (13.04 ± 5.38) × 10^6^ and (3.40 ± 1.68) × 10^6^ cells for bone marrow and spleen, respectively. For tumor-bearing mice, the neutrophil yield was (7.16 ± 2.92) × 10^6^, (1.12 ± 0.12) × 10^6^, and (1.36 ± 0.11) × 10^6^ cells per mouse for LLC, RLS_40_^Low^, and RLS_40_^High^, respectively. The purity of neutrophil samples was assessed by flow cytometry using anti-Ly6G antibodies (violetFluor 450, cat#ab253070, Abcam, Cambridge, UK) and was >95%.

### 2.7. RNA Isolation

RNA was isolated from bone marrow- and spleen-derived neutrophils using Rizol (diaGene, Moscow, Russia) according to the manufacturer’s instruction. The purity and integrity of the isolated RNA were analyzed using NanoDrop^®^ oneC (Thermo Fisher Scientific) and by gel electrophoresis.

### 2.8. Primer Design

Reference genes were selected after screening the literature. The RealTime PCR Tool was used to design the primers and probes (supported by Integrated DNA Technologies, https://eu.idtdna.com/scitools/Applications/RealTimePCR/, accessed on 10 December 2023) (Table 1). The self- and heterodimerization potentials of the primers and probes were analyzed using the OligoAnalyzer™ Tool (supported by Integrated DNA Technologies, https://eu.idtdna.com/pages/tools/oligoanalyzer, accessed on 10 December 2023). The amplicons were analyzed for secondary structures using MFOLD [51] (http://www.unafold.org/mfold/applications/dna-folding-form.php, accessed on 10 December 2023). Primers and probes were synthesized by the Syntol company (Moscow, Russia) or at the Laboratory of Biomedical Chemistry of ICBFM SB RAS (Novosibirsk, Russia).

### 2.9. cDNA Preparation and RT-qPCR

cDNA was prepared using reverse transcriptase M-MuLV–RH (Biolabmix, Novosibirsk, Russia). The reaction was carried in 40 μL which contained 2 μg of total RNA, 200 U reverse transcriptase in RT buffer (Biolabmix, Novosibirsk, Russia), and 1 μM of dT_18_ primers and 1 μM of random hexamers (ICBFM SB RAS, Novosibirsk, Russia). The reaction was carried at 42 °C for 1 h and terminated by heating at 70 °C for 10 min. Reverse-transcribed RNA samples were 10-fold diluted and stored in aliquots at −80 °C.

The reaction mixture for qPCR (12.5 μL) contained 12.5 ng of cDNA, BioMaster HS-qPCR SYBR (BiolabMix), and 0.4 μM of each of the forward and reverse specific primers and 0.25 μM of probes. The qPCR profile used was 95 °C for 6 min followed by 45 cycles of 95 °C for 15 s; 56 °C for 20 s, and 70 °C for 60 s. The amplification efficiency of primers was estimated using 1, 10, 100, and 1000-fold dilutions of pooled cDNAs of three technical replicates for each gene.

### 2.10. Gene Expression Stability Analysis

To study the expression stability of selected reference genes, we used BestKeeper [37], NormFinder [38], geNorm [39] and ΔCt method [40]. In addition, RefFinder was used to introduce an overall ranking [34]. BestKeeper was used in the Excel-integrated software downloaded from https://www.gene-quantification.de/bestkeeper.html (version 1, accessed on 31 July 2024). NormFinder was used in R applied by the authors from https://www.moma.dk/software/normfinder (accessed on 31 July 2024). The analysis of geNorm and the ΔCt method was conducted using the RefFinder tool at https://www.ciidirsinaloa.com.mx/RefFinder-master/ (accessed on 25 August 2024).

### 2.11. Validation of Selected Reference Genes

The relative fold change in the expression of the target gene *Arg1* was analyzed using the 2^−∆∆Ct^ method [52], employing *Tbp* or *Rpl13a* for normalization. The 2^−∆∆Ct^ was calculated in Microsoft Excel 365.

### 2.12. Data Analysis

Figures and descriptive statistics were constructed using GraphPad Prism version 8.0.2. or using R version 4.4.1.

## 3. Results

### 3.1. Experimental Design

**Figure 1:**
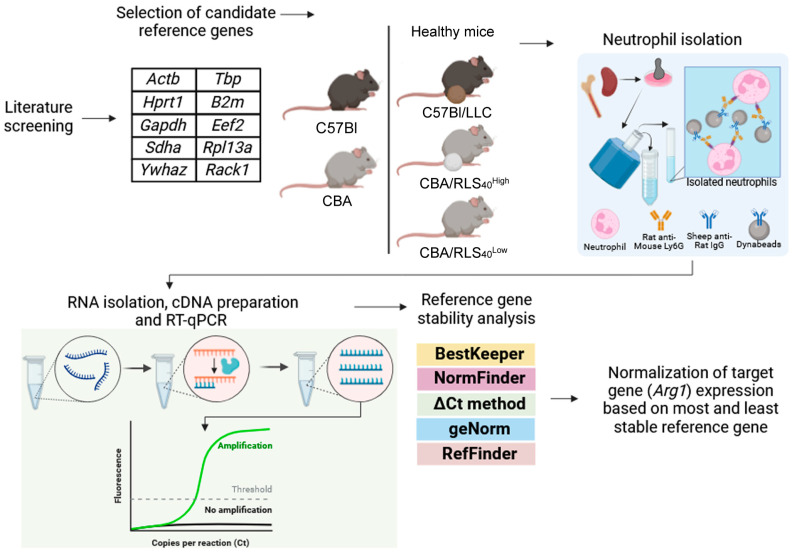
Experimental design.

The reference genes employed in this study were selected based on an analysis of literature data. Because few studies have focused on good reference genes for murine neutrophils, reference genes suggested for neutrophils in different species were chosen. Common reference genes used in murine studies were also used. To ensure the diversity of the selected reference genes, we focused on selecting reference genes with distinct biological functions (Table S1). The genes analyzed in this study and their functions are listed in Figure 1 and Table S1. Orthologs of *Actb*, *Hprt1*, *Sdha*, *Tbp*, *B2m*, and *Rack1* have been suggested as good housekeeping genes in human neutrophils [46,47]. *Ywhaz* has been suggested as a good reference gene in human leukocytes [39]. Moreover, *Ywhaz* is a good reference gene in bovine and sheep neutrophils [42,43,44,45]. The ortholog of *Rpl13a* is a good reference gene in bovine neutrophils [43]. *Eef2* is a constantly expressed housekeeping gene in all murine tissues without significant differences in expression levels [41]. *Gapdh* is a well-established reference gene and is one of the most commonly used reference genes [53,54].

Expression of selected reference genes was evaluated in bone marrow- and spleen-derived neutrophils of healthy mice and in spleen-derived neutrophils from mice with tumors of different histological genesis and metastasis pathways, LLC and RLS_40_. LLC is a reproducible and well-established syngeneic murine model of lung cancer [55]. LLC has an epithelial origin, metastasizes to the lungs, and is comparable to human lung cancer. LLC models are created using immunocompetent C57Bl mice; thus, the true immune responses can be evaluated with respect to tumor growth [56]. RLS_40_ was developed from lymphosarcoma susceptible to chemotherapy [57]. It corresponds to chemotherapy-resistant lymphosarcoma, whose resistance is developed in response to several courses of chemotherapy. RLS_40_ has a hematopoietic origin, metastasizes to the liver, and is comparable to malignant human lymphoma [58]. RLS_40_ is also created in immunocompetent mice, which allows us to evaluate the neutrophil profile as it exists during tumor development.

The experimental scheme is presented in Figure 1. LLC and RLS_40_ solid tumors were induced in C57Bl and CBA mice by intramuscular (i.m.) inoculation with LLC and RLS_40_ cells (10^6^ cells/mouse), respectively. On day 15 after LLC transplantation, spleens were harvested. On day 21, the mice with RLS_40_ were divided into two groups depending on the size of tumor nodes: RLS_40_^High^ mice with a tumor node size ≥1 cm^3^ and RLS_40_^Low^ mice with a tumor node size ≤ 0.1 cm^3^. The spleens of these mice were then harvested. Bone marrow and spleens of healthy mice C57Bl and CBA were used as a source of control neutrophils. Neutrophils were isolated by immunomagnetic positive selection; total RNA was isolated and used for RT-qPCR (see Materials and Methods). BestKeeper, NormFinder, ΔCt method, geNorm, and RefFinder were used to analyze the stability of the reference genes.

### 3.2. Histological Analysis of the Primary Tumor Node and Organs with Metastasis

**Figure 2:**
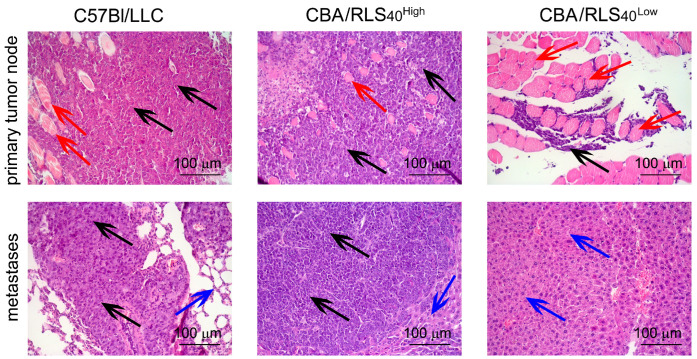
Histological analysis of primary tumor nodes and metastases in mice with LLC and RLS_40_. Hematoxylin and eosin staining. Original magnification: 200×. Black arrows indicate tumor cells. Red arrows indicate muscle fibrils. Blue arrows indicate lung or liver tissue.

Tumor development was controlled by histological analysis of the tumor implantation site (thigh muscles) and organs where metastasis occurs (lungs in the case of LLC and liver in the case of RLS_40_). LLC primary tumor nodes were presented by the polymorphic atypical epithelioid cells with large nuclei, coarse-grained chromatin, and wide cytoplasm (Figure 2). In some LLC cells, mitotic figures were observed (3–4 per field of view). In RLS_40_^High^ primary tumor nodes, large monomorphic atypical lymphoid cells with hyperchromic nuclei, narrow cytoplasmic rims, and a high mitotic rate were observed (4–8 per field of view).

Proliferation of the primary tumor nodes of both LLC and RLS_40_^High^ is characterized by expansive growth and thigh muscle destruction (Figure 2). In the stroma of both tumors, foci of necrosis with perifocal inflammatory infiltration were detected; however, destructive changes were more pronounced in RLS_40_ tissue. LLC and RLS_40_^High^ tumors give metastatic seeds to the lungs and liver, respectively, and their histological structure is similar to that of the relevant primary tumor node (Figure 2).

RLS_40_^Low^ tumor development is accompanied by the resorption of palpable masses in the thigh muscles, the presence of only individual tumor cells between muscle fibrils, and the absence of metastatic lesions in the liver that can be explained by tumor cell eradication due to immune surveillance (Figure 2).

### 3.3. Gene Expression Profile of Selected Reference Genes

The experimental groups used in the study are listed in Table 2.

In tumor models, splenic neutrophils from mice with LLC (n = 3), mice with RLS_40_^High^ (n = 3) and mice with RLS_40_^Low^ (n = 3) were investigated. As a control, bone marrow- and spleen-derived neutrophils of healthy mice from the corresponding mouse strains (C57Bl and CBA, respectively, n = 3) were used (Table 2).

The efficiencies of the primer pairs were calculated to ensure the comparability between RT-qPCR results. The efficiencies were between 90.29% and 104.39% (Table S1), which are considered in the goal efficiency interval (90–110%) [59]. Moreover, the correlation coefficient R^2^ was between 0.9933 and 0.9949, indicating the low variability across assay replicates (Table S1) [59].

**Figure 3:**
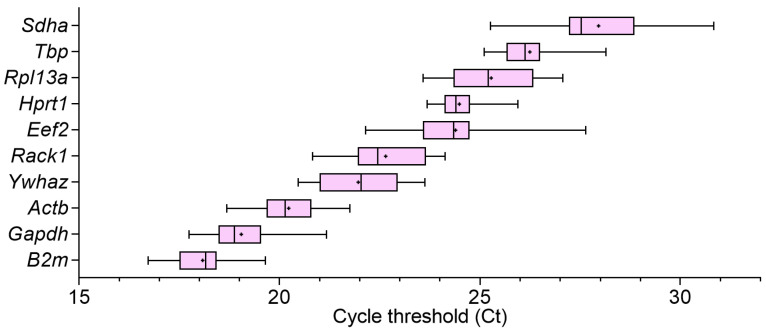
RT-qPCR cycle threshold values of selected reference genes in bone marrow-derived and spleen-derived neutrophils from healthy and tumor-bearing mice (21 samples: spleen-derived neutrophils from mice with LLC (n = 3), from mice with RLS_40_^High^ (n = 3), from mice with RLS_40_^Low^ (n = 3), and bone marrow-derived and spleen-derived neutrophils of healthy mice from the corresponding mouse strains (C57Bl and CBA, respectively, n = 3)). The boxes show the interquartile interval between the 25th and 75th percentiles, and the whiskers show the min-to-max interval. Crossbar indicates the median, and the + symbol indicates the mean.

The Ct values for all reference genes were in the applicable range < 40 (16.73–30.83) (Figure 3), which ensured the applicability of the quantification results. The highest expression levels were observed for *B2m* (18.08 ± 0.70), while the lowest expression levels were observed for *Sdha* (27.96 ± 1.29) (Figure 3).

Unlike the overall SD values and the distribution of Ct values across the entire sample population (Figure 3), the distribution of Ct values within each group illustrated the differences in expression patterns among the different groups (Figures S1 and S2). Some genes, like *Hprt1*, showed relatively similar expression patterns in the studied groups with relatively low SD values (Figures S1B and S2B). Other genes, such as *Gapdh* and *Sdha*, showed relatively high variability within each studied group (Figures S1C,D and S2C,D). *Ywhaz* and *Tbp* expression was relatively higher in the spleen than in bone marrow samples (Figures S1E,F and S2E,F). For some genes higher expression variability was noticed in the RLS40^High^ samples (Figure S2D,F,H,J). In the case of *Rpl13a*, there was a notable increase in the gene expression in splenic neutrophils from tumor-bearing mice (Figures S1I and S2I). Similar trends were observed for *B2m* (Figures S1G and S2G). This differential expression pattern could indicate the instability of the reference gene due to neutrophil activation after tumor development.

### 3.4. Gene Stability Analysis

To study the expression stability of selected reference genes, BestKeeper [37], NormFinder [38], geNorm [39] and ΔCt method [40] were used (Figure 1). In addition, RefFinder was used to introduce an overall ranking [34].

#### 3.4.1. BestKeeper Results

The principle of BestKeeper is that a stably expressed reference gene should exhibit low variation in expression levels across different samples from different experimental groups. In BestKeeper, the ranking of reference genes is determined by the standard deviation (SD) and coefficient of variation (CV) of their expression levels, with the genes that are most stably expressed showing the least variation, while the least stable genes show the most variation. Any studied gene with SD > 1 can be considered inconsistent [37]. In addition, BestKeeper provides descriptive analysis of the studied sample set, including geometric and arithmetic means of the Ct values of the reference genes and min and max values of the Ct values. As mentioned previously, *B2m* expression was the highest, whereas *Sdha* expression was the lowest (Table 3). The reference genes with the lowest variation were *Hprt1*, *B2m*, and *Tbp*. *Eef2*, *Rpl13a*, and *Sdha* exhibited the highest variation (Table 3). All SD values of reference gene expression were <1 except for *Sdha* (SD = 1.02). The statistics provided by BestKeeper and the gene rankings from most to least stable (from 1 to 10) are presented in Table 3.

#### 3.4.2. NormFinder Results

**Figure 4:**
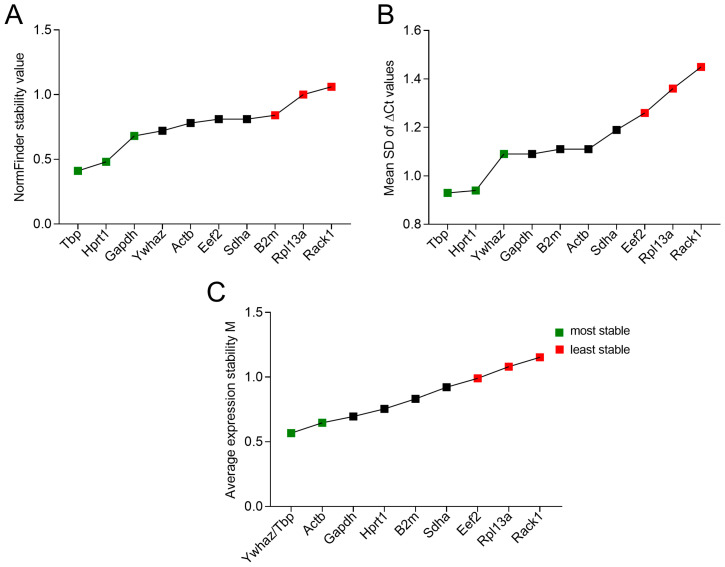
Expression stability of reference genes. (**A**) NormFinder analysis. (**B**) ΔCt method. (**C**) geNorm analysis.

To integrate the intergroup expression variation of the reference genes studied, we applied NormFinder [38]. The sample set was divided into subgroups according to the source of neutrophils (bone marrow or spleen), mouse strain (C57Bl or CBA), and transplanted tumor model (no tumor, LLC, RLS_40_^High^, RLS_40_^Low^). The results are shown in Figure 4A. *Tbp* and *Hprt1* were the most stably expressed genes across the sample set (Table S2, Figure 4A). Moreover, we tried to divide the sample set into a more simplified approach, i.e., healthy or tumor groups. The overall ranking somehow changed; however, *Tbp* and *Hprt1* remained the most stably expressed reference genes (Table S3).

#### 3.4.3. ΔCt Results

The ΔCt method is based on calculating ΔCt for every pair of studied reference genes and calculates the SD of ΔCt gained for all samples. The genes are then ranked based on the average of the SDs of ΔCt values gained for every gene from all possible gene pair combinations [40]. The lower the average of the SD of ΔCt values, the more stable the gene.

The results showed that *Tbp* and *Hprt1* were the most stable genes with a stability value < 1 (Figure 4B, Table S4). The least stable genes were *Rpl13a* and *Rack1* (Figure 4B, Table S4).

#### 3.4.4. geNorm Results

geNorm calculates the pairwise variation of a reference gene against all other reference genes by measuring the standard deviation of the logarithmically transformed expression ratios and establishes the internal reference gene stability measure M as the average pairwise variation of a specific gene with all other reference genes [39]. The algorithm finds two genes that show the most agreement in expression and subsequently determines the expression variation of all other genes in relation to these two selected ones. Consequently, the algorithm always suggests two genes at the top with the lowest M value followed by additional genes that have greater M values and lower stability [39,60]. Based on the M values of the studied genes, geNorm suggested *Ywhaz*, *Tbp* as the most stable pair, whereas *Eef2*, *Rpl13a*, and *Rack1* were the least stable reference genes (Figure 4C, Table S5). Because *Ywhaz* exhibited high variation in the studied sample set (Table 3), we hypothesized that *Ywhaz* could be correlated with *Tbp* because the method could confirm that co-regulated and correlated genes were stable [60]. To prove this hypothesis, we performed Pearson’s correlation on the linearized Ct values for all genes and samples. Interestingly, a high correlation was observed between *Ywhaz* and *Tbp*, with the highest Pearson’s r score of all genes of 0.87, which could explain the observed opposite findings (Table S6, Figure S3). Another pair with a Pearson’s r score of 0.87 is *Actb* and *Gapdh*, falling directly after *Ywhaz*/*Tbp* in the ranking.

#### 3.4.5. Comprehensive Ranking of Reference Genes

To summarize the observed findings, we used RefFinder, which assigns weights to each reference gene according to the ranking of each of the four algorithms used and calculates the geometric mean of the weights from each ranking [34]. The algorithm of RefFinder ensures that each used method (BestKeeper, NormFinder, ΔCt method and geNorm) contributes equally to the final overall ranking. Using RefFinder, *Tbp* and *Hprt1* were identified as potential reference genes for splenic neutrophils (Table 4 and Table S7). *Rack1* and *Rpl13a* were the least stably expressed reference genes in the studied cells (Table 4 and Table S7).

### 3.5. RT-qPCR Normalization Using Different Reference Genes

**Figure 5:**
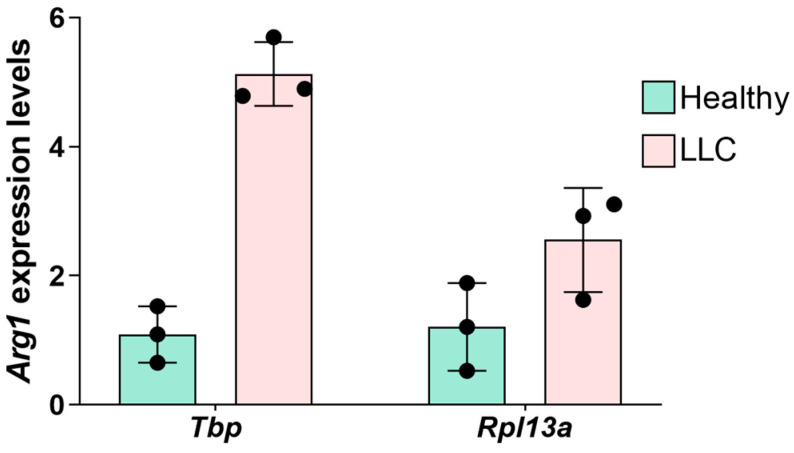
RT-qPCR normalization based on *Tbp* or *Rpl13a* affects the result interpretation of expression levels of *Arg1* in splenic neutrophils isolated from healthy or LLC-bearing mice (n = 3). The results are expressed as fold change in expression relative to healthy control. Data are shown in mean ± SD.

In order to show how reference gene selection could affect the gene expression results, we studied the expression of *Arg1* in splenic neutrophils isolated from healthy or LLC-bearing mice and normalized the results to the most stably expressed reference gene *Tbp* and the least stably expressed gene *Rpl13a* (Table 4). We used the 2^−ΔΔCt^ method to compare the expression of *Arg1* when normalized to *Tbp* or to *Rpl13a* [52]. A 5-fold change in *Arg1* expression was observed, when data were normalized to *Tbp* (Figure 5). However, this fold change decreased to 2.5 when the less stable reference gene *Rpl13a* was used (Figure 5).

## 4. Discussion

When using RT-qPCR, it is imperative to select an appropriate reference gene for data normalization. The use of an inappropriate reference gene can lead to misleading analysis of RT-qPCR data. A suitable reference gene must be stably expressed across all studied samples and should not be affected by any experimental conditions or treatment. In addition, the reference gene should be expressed stably regardless of the activation state of the studied cell population. This is especially important for immune cells because their activation or maturation states can affect the stability of reference gene expression [28].

The issue of reference gene selection for neutrophils is particularly important for several reasons: first, the robust changes in the neutrophil transcriptome in response to minor environmental cues [32]. Second, the unsuitability of some commonly used reference genes because they can be upregulated under cellular activation. For example, β-actin, the product of *Actb*, controls cell motility, which is an important feature of neutrophils under activation [61]. Moreover, it controls the G-actin ratio in the cell, which is an important aspect in neutrophil activation and NETosis [61,62].

Under pathological conditions, including tumor development, splenic neutrophils are activated, and the expression profile of some neutrophil genes significantly change [10,16,17,18,19,20,63]. It has been noted that neutrophils accumulate in the spleen during tumor development in mice [64,65]. The activation profile of splenic neutrophils can be influenced by various stimuli generated by the tumor and the tumor environment [66]. Several studies have identified reference genes for neutrophils of humans and other mammals [42,43,44,45,46,47]; however, a clear lack of data on reference genes for mouse neutrophils was noted. To fill this gap, the aim of this study was to identify a stably expressed reference gene in murine splenic neutrophils isolated from healthy mice and mice with tumors.

The investigated reference genes *Tbp*, *Hprt1*, *Ywhaz*, *B2m*, *Gapdh*, *Actb*, *Sdha*, *Eef2*, *Rack1*, and *Rpl13a* were chosen based on the screening of reference genes for neutrophils from different species [39,41,42,43,44,45,46,47,53,54]. We focused on including reference genes that encode proteins from different families with distinct functions to ensure the diversity and minimize the co-regulation of the studied genes [40] (Table S1).

As a tumor model, we selected LLC and RLS_40_. LLC, first isolated in 1951 by Dr. Margaret Lewis, is a reproducible and well-established syngeneic murine model that metastasizes into the lungs [55]. RLS_40_ developed from lymphosarcoma that is susceptible to chemotherapy has a hematopoietic origin, metastasizes to the liver, and is comparable to malignant human lymphoma [57]. RLS_40_ was used to evaluate reference genes for splenic neutrophils of mice with a different immune status, including mice with a tumor node size ≥1 cm^3^ (RLS_40_^High^), which corresponds to the tumor escaping from immune surveillance, and mice with a tumor node size ≤ 0.1 cm^3^ (RLS_40_^Low^) that corresponds to tumor growth controlled by the immune system (Figure 1, Table 2). In these two models, splenic neutrophils could have different profiles and functional states than spleen- and bone-marrow-derived neutrophils from healthy mice (control). Therefore, if reference genes are expressed in the same manner in all neutrophil states, then they can be considered appropriate stably expressed reference genes.

**Figure 6:**
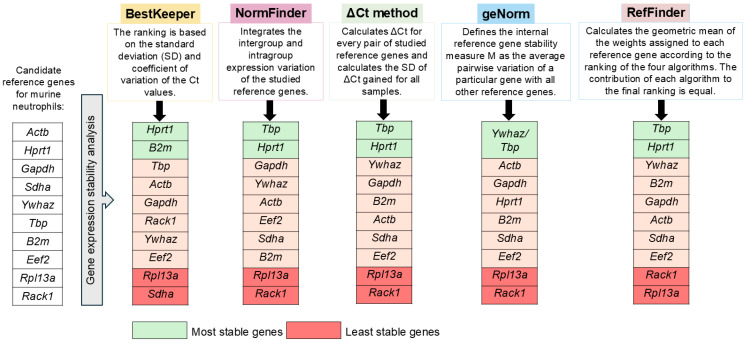
Principles of the instruments used in this study and the ranking of the investigated reference genes in murine neutrophils.

It is not surprising that different programs gave different reference gene rankings because of the different principles of working of these algorithms, which makes understanding each algorithm’s pitfalls necessary when analyzing and comparing the different gene rankings (Figure 6). The results of BestKeeper are based on the overall variances of separate genes and it is the only method in which genes do not influence the ranking of others. Regarding SD values of Ct, *Hprt1*, *B2m*, and *Tbp* had the least variance among all genes (Table 3, Figure 6).

However, in our experimental design, it was a necessity to apply NormFinder, a more robust tool that considers intergroup variance, to ensure that the chosen reference gene shows minimal variation not just “overall expression variation”, but also minimal variation across the sample sub-groups. The selected subgroups represent neutrophils in the steady or activated state, which is an important factor to consider when selecting reference genes for immune cells [28]. When using NormFinder, *Tbp* and *Hprt1* remained the genes with the highest stability, whereas *B2m* lost its ranking mostly because of high intergroup variance (Figure 4A, Tables S2 and S3). This finding aligns with the previous observation of the differential pattern of *B2m* expression in healthy vs. tumor groups (Figures S1G and S2G), highlighting the influence of tumor development on the expression stability of *B2m*.

Pairwise methods (geNorm and ΔCt) can produce misleading conclusions when genes in the gene set are correlated [60]. This was observed when a gene with high variability in the sample set *Ywhaz* was ranked as one of the most stable genes according to ΔCt method and was in the top pair according to geNorm (Figure 4B,C, Tables S4 and S5). This could be explained by the high correlation between *Tbp* and *Ywhaz* observed (Table S6, Figure S3), which is a limitation in the use of geNorm. This correlation could be explained by the similar pattern of expression of these two genes (Figures S1E,F and S2E,F). However, in the case of the ΔCt method, there was no correlation with the top two genes (*Tbp* and *Hprt1*) with a Pearson’s r score 0.34 (Table S6). Moreover, *Hprt1* expression was not correlated with any of the other studied genes and exhibited low variation across the studied samples (Table S6, Figure S3). However, *Ywhaz* could be overestimated in the ΔCt algorithm, as in the geNorm algorithm, because of the possible correlation with *Tbp* (Table S6, Figure S3).

In our sample set, using RefFinder was helpful to take a comprehensive look at the overall ranking, although its results could be confusing in another more heterogeneous set because it is based solely on the geometric mean of the rankings. In our case, RefFinder suggested that as was the case with most of the other algorithms, *Tbp* and *Hprt1* were the most stably expressed reference genes in murine splenic neutrophils, whereas *Rack1* and *Rpl13a* were the least stably expressed genes (Table 4 and Table S7, Figure 6).

Using different algorithms, among the 10 investigated reference genes, we identified *Tbp* and *Hprt1* as appropriate reference genes for murine bone marrow-derived and splenic neutrophils (Table 4 and Table S7, Figure 6).

It is important to note that commonly used reference genes like *Gapdh* and *Actb* are not appropriate for murine neutrophils and exhibit relatively low gene expression stability with high variability between samples (Table 3 and Table 4, Figure 6). This highlights the necessity of overlooking the traditionally used reference genes, especially when investigating immune cells.

As a type of validation, the expression of *Arg1* in splenic neutrophils from healthy or LLC-bearing mice, normalized to *Tbp* or *Rpl13a*, was studied (Figure 5). *Arg1* was selected because its expression is known to be significantly increased in neutrophils during tumor development, especially in tumor-associated neutrophils [67,68]. It was shown that the fold change elevation of *Arg1* expression was reduced to half when the inappropriate reference gene *Rpl13a* was used for normalization (Figure 5).

To the best of our knowledge, earlier published articles on identifying reference genes for murine neutrophils are lacking. Because of the wide range of investigated populations in this study, we suggest that these results could be applicable to splenic neutrophils isolated from different murine tumor models. Moreover, the suggested reference genes could be applicable to murine blood or bone marrow-derived neutrophils.

## 5. Conclusions

In conclusion, we emphasize the importance of selecting reference genes for neutrophils rather than commonly used reference genes. We highlight the importance of understanding the algorithms of existing programs for analyzing gene expression stability to provide rational suggestions for comprehensively ranking the studied reference genes. We conclude that *Tbp* and *Hprt1* are appropriate reference genes for murine splenic neutrophils.

### Limitations

Our study has some potential limitations. The candidate reference genes were selected on the basis of literature screening for already identified reference genes appropriate for human neutrophils and neutrophils from other species. Despite the relatively low variability of the top selected reference genes in our study, other undiscovered more suitable gene candidates could have been spontaneously excluded. Further screening of published neutrophil transcriptome sequencing data may help identify potential stable reference genes for the cell population under study [69,70,71,72,73].

## Figures and Tables

**Table 1:** List of primers and probes used in the study.

Gene	Sequences of Primers and Probes, 5′ → 3′		Amplicon Size, bp
*Actb*	F	TATTGGCAACGAGCGGTTCC	140
	R	TGGCATAGAGGTCTTTACGG	
	P	((5,6)-ROX)-CCAGCCTTCCTTCTTGGGTATGGAATCC-BHQ2	
*Hprt1*	F	CCCCAAAATGGTTAAGGTTGC	76
	R	AACAAAGTCTGGCCTGTATCC	
	P	((5,6)-ROX)-CTTGCTGGTGAAAAGGACCTCTCGAA-BHQ2	
*Gapdh*	F	CAAGGAGTAAGAAACCCTGGAC	109
	R	GGATGGAAATTGTGAGGGAGAT	
	P	((5,6)-ROX)-CCAGCAAGGACACTGAGCAAGAGA-BHQ2	
*Sdha*	F	CCTACCCGATCACATACTGTTG	73
	R	AGTTGTCCTCTTCCATGTTCC	
	P	((5,6)-ROX)-CAGAGCAGCATTGATACCTCCCTGT-BHQ2	
*Ywhaz*	F	GAAGACGGAAGGTGCTGAG	148
	R	GACTTTGCTTTCTGGTTGCG	
	P	((5,6)-ROX)-AGAGAGAAGATCGAGACGGAGCTGC-BHQ2	
*Tbp*	F	AAGAAAGGGAGAATCATGGACC	133
	R	GAGTAAGTCCTGTGCCGTAAG	
	P	((5,6)-ROX)-CCTGAGCATAAGGTGGAAGGCTGTT-BHQ2	
*B2m*	F	GGTCGCTTCAGTCGTCAG	150
	R	TTCAGTATGTTCGGCTTCCC	
	P	((5,6)-ROX)-CCCTGGTCTTTCTGGTGCTTGTCT-BHQ2	
*Eef2*	F	ACATTCTCACCGACATCACC	135
	R	GAACATCAAACCGCACACC	
	P	((5,6)-ROX)-GAACATCAAACCGCACACC-BHQ2	
*Rpl13a*	F	CAAGACCAACGGACTCCTG	146
	R	TCTCTAATGTCCCCTCTACCC	
	P	((5,6)-ROX)-AAGACTGTTTGCCTCATGCCTGC-BHQ2	
*Rack1*	F	AATACTCTGGGTGTCTGCAAG	146
	R	TTAGCCAGATTCCACACCTTG	
	P	((5,6)-ROX)-ATGGGTGTCTTGTGTCCGCTTCTC-BHQ2	
*Arg1*	F	AAGAATGGAAGAGTCAGTGTGG	132
	R	GGGAGTGTTGATGTCAGTGTG	
	P	((5,6)-FAM)-TCTGGCAGTTGGAAGCATCTCTGG-BHQ1	

**Table 2:** Experimental groups used in the study.

Experimental Group	Mice Strain, n	Source of Neutrophils
Healthy	C57Bl, 15	Bone marrow and spleen
Healthy	CBA, 15	Bone marrow and spleen
LLC	C57Bl, 15	Spleen
RLS_40_^High^	CBA, 15 *	Spleen
RLS_40_^Low^	CBA, 15 *	Spleen

*—number of mice included in the experiment according to tumor node size criteria. RLS_40_^High^—tumor node size ≥ 1 cm^3^; RLS_40_^Low^—tumor node size ≤ 0.1 cm^3^.

**Table 3:** Descriptive statistics and stability analysis calculated by BestKeeper.

	*Actb*	*Hprt1*	*Gapdh*	*Sdha*	*Ywhaz*	*Tbp*	*B2m*	*Eef2*	*Rpl13a*	*Rack1*
Geo Mean [CP]	20.21	24.48	19.03	27.93	21.94	26.23	18.07	24.36	25.26	22.63
Ar Mean [CP]	20.23	24.48	19.05	27.96	21.97	26.24	18.08	24.39	25.28	22.65
min [CP]	18.69	23.68	17.74	25.26	20.47	25.10	16.73	22.15	23.58	20.82
max [CP]	21.76	25.94	21.17	30.83	23.63	28.14	19.65	27.64	27.07	24.13
SD [±CP]	0.63	0.35	0.63	1.02	0.88	0.57	0.55	0.94	0.95	0.81
CV [% CP]	3.13	1.43	3.33	3.64	3.98	2.18	3.07	3.87	3.74	3.59
Ranking [1–>10]	4	1	5	10	7	3	2	8	9	6

n = 21; CP—crossing point; Geo—geometric; Ar—arithmetic; SD—standard deviation; CV—percentage coefficient of variation.

**Table 4:** Overall ranking of reference genes according to the employed algorithms (BestKeeper, NormFinder, ΔCt method and geNorm) and comprehensive ranking calculated by RefFinder.

Rank ^†^	BestKeeper	NormFinder	ΔCt Method	geNorm	RefFinder
1	*Hprt1*	*Tbp*	*Tbp*	*Ywhaz/Tbp*	*Tbp*
2	*B2m*	*Hprt1*	*Hprt1*		*Hprt1*
3	*Tbp*	*Gapdh*	*Ywhaz*	*Actb*	*Ywhaz*
4	*Actb*	*Ywhaz*	*Gapdh*	*Gapdh*	*B2m*
5	*Gapdh*	*Actb*	*B2m*	*Hprt1*	*Gapdh*
6	*Rack1*	*Eef2*	*Actb*	*B2m*	*Actb*
7	*Ywhaz*	*Sdha*	*Sdha*	*Sdha*	*Sdha*
8	*Eef2*	*B2m*	*Eef2*	*Eef2*	*Eef2*
9	*Rpl13a*	*Rpl13a*	*Rpl13a*	*Rpl13a*	*Rack1*
10	*Sdha*	*Rack1*	*Rack1*	*Rack1*	*Rpl13a*

^†^ From most stable to least stable.

## Data Availability

The raw data supporting the conclusions of this article will be made available by the corresponding author (N.L.M.) upon e-mail request.

## Supplementary Materials

The following supporting information can be downloaded at https://www.mdpi.com/article/10.3390/biomedicines12112571/s1: Figure S1: Ct values of reference genes in neutrophils isolated from the spleens of LLC-bearing mice compared with bone marrow (BM) and spleen-derived neutrophils isolated from corresponding healthy mice (C57Bl/6). Data are shown as mean ± SD. Kruskal–Wallis test with Dunn’s multiple comparisons test was used. Figure S2: Ct values of reference genes in neutrophils isolated from spleens of RLS40-bearing mice in comparison to bone marrow (BM) and spleen-derived neutrophils isolated from corresponding healthy mice (CBA). Data are shown as mean ± SD. Kruskal–Wallis test with Dunn’s multiple comparisons test was used. No statistical significance (*p* < 0.05) was found. Figure S3: Pearson’s correlation heatmap of the linearized Ct values (2^−Ct^) of all genes; Table S1: Selected reference gene functions and amplification efficiency parameters; Table S2: Intergroup and intragroup variation and stability values of reference gene expression calculated with NormFinder. The subgroups are based on the source of neutrophils (bone marrow or spleen), mouse strain (C57Bl or CBA) and on the transplanted tumor model (no tumor, LLC, RLS_40_^High^, RLS_40_^Low^); Table S3: NormFinder results of reference gene expression stability based on subdividing the sample set into healthy or tumor neutrophils; Table S4: Gene stability calculated by ΔCt method; Table S5: Gene stability calculated by geNorm; Table S6: Pearson’s correlation matrix of the linearized Ct values (2^−Ct^) of the studied genes; Table S7: Comprehensive ranking calculated by RefFinder.
