# Supplementary material for: Tbp and Hprt1 Are Appropriate Reference Genes for Splenic Neutrophils Isolated from Healthy or Tumor-Bearing Mice
Source: Biomedicines. 2024 Nov 10;12(11):2571. doi: 10.3390/biomedicines12112571 (PMC11592060; doi:10.3390/biomedicines12112571)
Supplement: Supplementary file 1 [file biomedicines-12-02571-s001.zip › biomedicines-3275734-supplementary.pdf]

Supplemental Material to: *Tbp* and *Hprt1* Are Appropriate Reference Genes for Splenic Neutrophils Isolated from Healthy or Tumor-Bearing Mice

Khetam Sounbuli [1,2], Ludmila A. Alekseeva [1], Aleksandra V. Sen'kova [1], Innokenty A. Savin [1], Marina A. Zenkova [1] and Nadezhda L. Mironova [1,*]

[1] Institute of Chemical Biology and Fundamental Medicine SB RAS, Lavrentiev Ave., 8, Novosibirsk 630090, Russia
[2] Faculty of Natural Sciences, Novosibirsk State University, Pirogova St., 1, Novosibirsk 630090, Russia
* Correspondence: mironova@niboch.nsc.ru; Tel.: +7-(385)-3635161

Table S1. Selected reference gene functions and amplification efficiency parameters.

Gene	Full name and function*	RT-qPCR efficiency, %[§]	Correlation coefficient (R^2)
Actb	actin beta, involved in cell motility, structure, integrity, and intercellular signaling	104.39	0.9933
Hprt1	hypoxanthine phosphoribosyl transferase 1, involved in the generation of purine nucleotides through the purine salvage pathway	103.36	0.9991
Gapdh	glyceraldehyde-3-phosphate dehydrogenase, involved in carbohydrate metabolism	97.24	0.9950
Sdha	succinate dehydrogenase complex flavoprotein subunit A, involved in the mitochondrial respiratory chain	102.21	0.9979
Ywhaz	tyrosine 3-monooxygenase/tryptophan 5-monooxygenase activation protein, mediates signal transduction by binding to phosphoserine-containing proteins	97.83	0.9960
Tbp	TATA-box binding protein, involved in the initiation of transcription by RNA polymerase II	94.06	0.9949
B2m	beta-2 microglobulin, MHC class II associated protein	99.5	0.9956
Eef2	eukaryotic translation elongation factor 2, GTP-binding translation elongation factor	94.81	0.9989
Rpl13a	ribosomal protein L13a, a component of the 60S subunit	96.84	0.9970
Rack1	receptor for activated C kinase 1, enables ion channel inhibitor activity	90.29	0.9938

* Provided by NCBI Gene portal
[§] Calculated by the standard curve method

Figure S1. Ct values of reference genes in neutrophils isolated from the spleens of LLC-bearing mice compared with bone marrow (BM) and spleen-derived neutrophils isolated from corresponding healthy mice (C57Bl/6). Data are shown as mean ± SD. Kruskal–Wallis test with Dunn's multiple comparisons test was used.

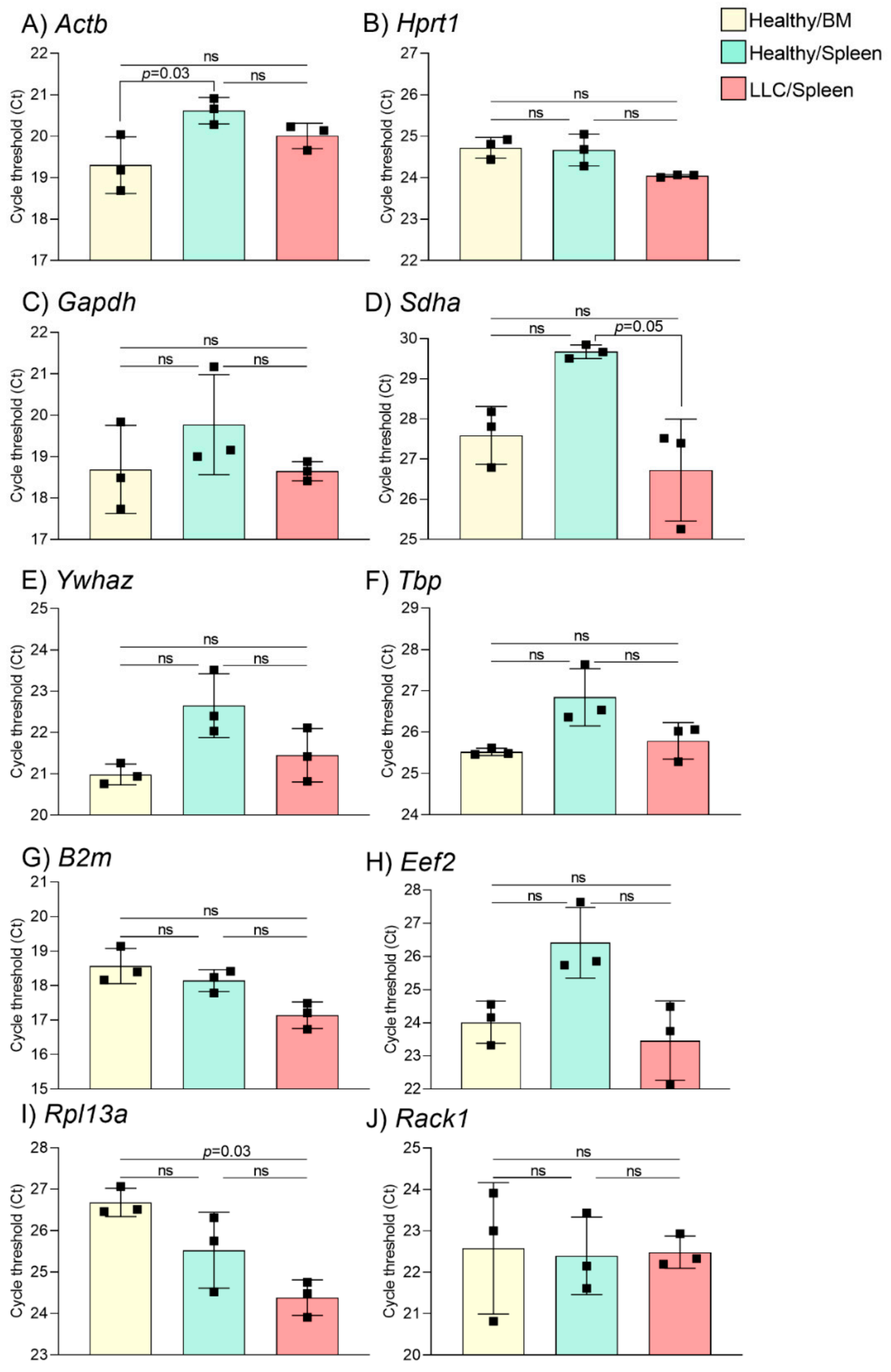

Figure S2. Ct values of reference genes in neutrophils isolated from spleens of RLS_{40}-bearing mice in comparison to bone marrow (BM) and spleen-derived neutrophils isolated from corresponding healthy mice (CBA). Data are shown as mean ± SD. Kruskal–Wallis test with Dunn's multiple comparisons test was used. No statistical significance ($p < 0.05$) was found.

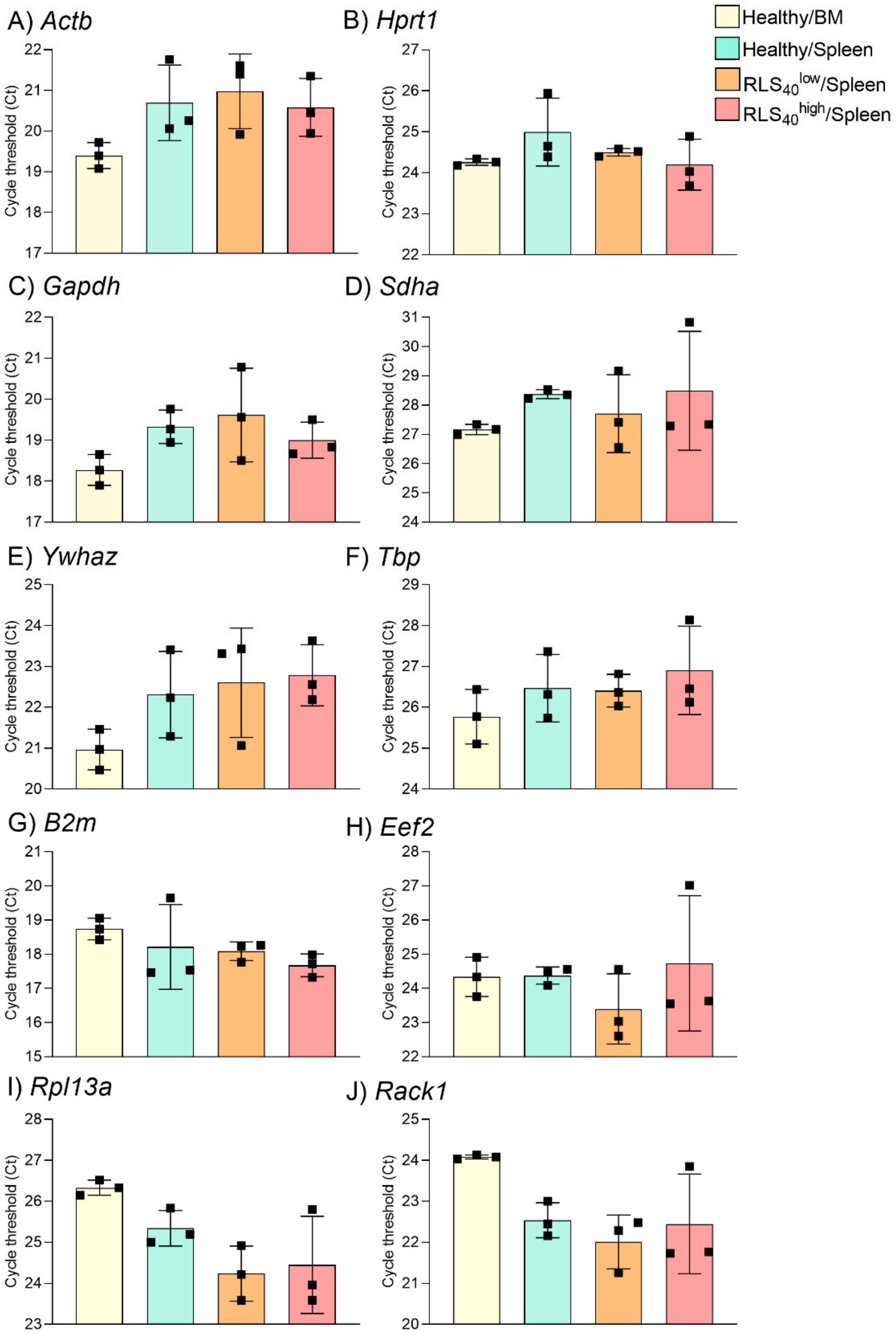

Table S2. Intergroup and intragroup variation and stability values of reference gene expression calculated with NormFinder. The subgroups are based on the source of neutrophils (bone marrow or spleen), mouse strain (C57bl or CBA) and on the transplanted tumor model (no tumor, LLC, RLS_{40}^{high}, RLS_{40}^{low}).

**Studied gene**	**Group difference***	**Group SD§**	**Stability value†**
Tbp	1.17	0.24	0.41
Hprt1	0.85	0.33	0.48
Gapdh	1.26	0.82	0.68
Ywhaz	1.77	0.51	0.72
Actb	1.46	0.70	0.78
Eef2	2.87	0.76	0.81
Sdha	2.22	0.73	0.81
B2m	2.50	0.94	0.84
Rpl13a	3.26	0.63	1.00
Rack1	3.10	1.11	1.06

*group difference is a measure of the difference between the groups

§ group SD is the common standard deviation within a group and stability value which combines both both the intra- and intergroup variation

† genes are listed with decreasing expression stability from top to bottom

Table S3. NormFinder results of reference gene expression stability based on subdividing the sample set into healthy or tumor neutrophils.

**Studied gene**	**Group difference***	**Group SD§**	**Stability value†**
Hprt1	0.1	0.39	0.21
Tbp	0.51	0.26	0.36
Sdha	0.26	0.9	0.45
B2m	0.06	1.13	0.46
Gapdh	0.39	0.75	0.46
Rack1	0.29	1.28	0.53
Eef2	0.66	0.95	0.6
Ywhaz	0.85	0.6	0.6
Actb	0.84	0.68	0.61
Rpl13a	1.34	0.93	0.82

*group difference is a measure of the difference between the groups

§ group SD is the common standard deviation within a group and stability value which combines both both the intra- and intergroup variation

† genes are listed with decreasing expression stability from top to bottom

Table S4. Gene stability calculated by ΔCt method.

Gene	The mean of SDs of ΔCt values†
Tbp	0.93
Hprt1	0.94
Ywhaz	1.09
Gapdh	1.09
B2m	1.11
Actb	1.11
Sdha	1.19
Eef2	1.26
Rpl13a	1.36
Rack1	1.45

† genes are listed with decreasing expression stability from top to bottom.

Table S5. Gene stability calculated by geNorm.

Gene	M value†
Ywhaz/Tbp	0.567
Actb	0.647
Gapdh	0.695
Hprt1	0.754
B2m	0.832
Sdha	0.922
Eef2	0.991
Rpl13a	1.080
Rack1	1.153

† genes are listed with decreasing expression stability from top to bottom.

Table S6. Pearson's correlation matrix of the linearized Ct values (2^{-Ct}) of the studied genes.

	Actb	*Hprt1*	*Gapdh*	*Sdha*	*Ywhaz*	*Tbp*	*B2m*	*Eef2*	*Rpl13a*	*Rack1*
Actb	1.00	0.00	0.87	0.35	0.83	0.69	-0.03	0.13	-0.55	-0.53
Hprt1	0.00	1.00	0.16	0.37	0.22	0.34	0.39	0.31	0.58	-0.07
Gapdh	0.87	0.16	1.00	0.44	0.77	0.60	0.06	0.22	-0.35	-0.67
Sdha	0.35	0.37	0.44	1.00	0.56	0.59	0.47	0.86	0.09	-0.16
Ywhaz	0.83	0.22	0.77	0.56	1.00	0.87	-0.01	0.34	-0.43	-0.41
Tbp	0.69	0.34	0.60	0.59	0.87	1.00	0.12	0.50	-0.13	-0.15
B2m	-0.03	0.39	0.06	0.47	-0.01	0.12	1.00	0.36	0.35	-0.20
Eef2	0.13	0.31	0.22	0.86	0.34	0.50	0.36	1.00	0.37	0.10
Rpl13a	-0.55	0.58	-0.35	0.09	-0.43	-0.13	0.35	0.37	1.00	0.39
Rack1	-0.53	-0.07	-0.67	-0.16	-0.41	-0.15	-0.20	0.10	0.39	1.00

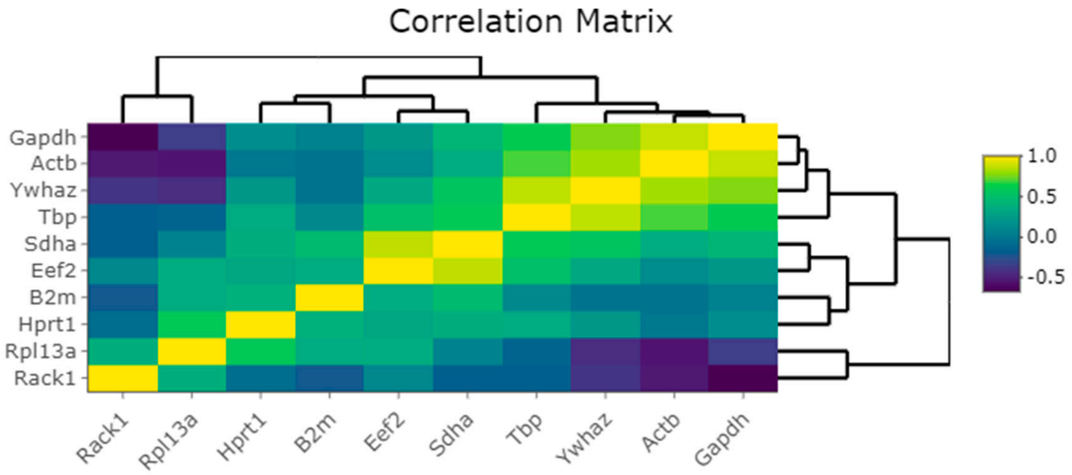

Figure S3. Pearson's correlation heatmap of the linearized Ct values (2^{-Ct}) of all genes. The numerical values used in the color scheme represent the Pearson's r score.

Table S7. Comprehensive ranking calculated by RefFinder.

gene	Geomean of ranking values[†]
Tbp	1.57
Hprt1	1.78
Ywhaz	3.20
B2m	3.66
Gapdh	4.23
Actb	4.56
Sdha	7.65
Eef2	8.00
Rack1	8.80
Rpl13a	9.00

[†] genes are listed with decreasing expression stability from top to bottom.
